# Supplementary material for: Vanilloid-dependent TRPV1 opening trajectory from cryoEM ensemble analysis
Source: Nat Commun. 2022 May 24;13:2874. doi: 10.1038/s41467-022-30602-2 (PMC9130279; doi:10.1038/s41467-022-30602-2)
Supplement: Supplementary file 1 — Supplementary Information [file 41467_2022_30602_MOESM1_ESM.pdf]

Supplementary Information

Vanilloid-dependent TRPV1 opening trajectory from cryoEM ensembles

Do Hoon Kwon[1], Feng Zhang[1], Justin G. Fedor[1], Yang Suo[1], Seok-Yong Lee[1,*]

Affiliations:
[1] Department of Biochemistry, Duke University School of Medicine, Durham, North Carolina, 27710, USA.

*Correspondence to:
S.-Y. Lee
Email: seok-yong.lee@duke.edu
Telephone: 919-684-1005

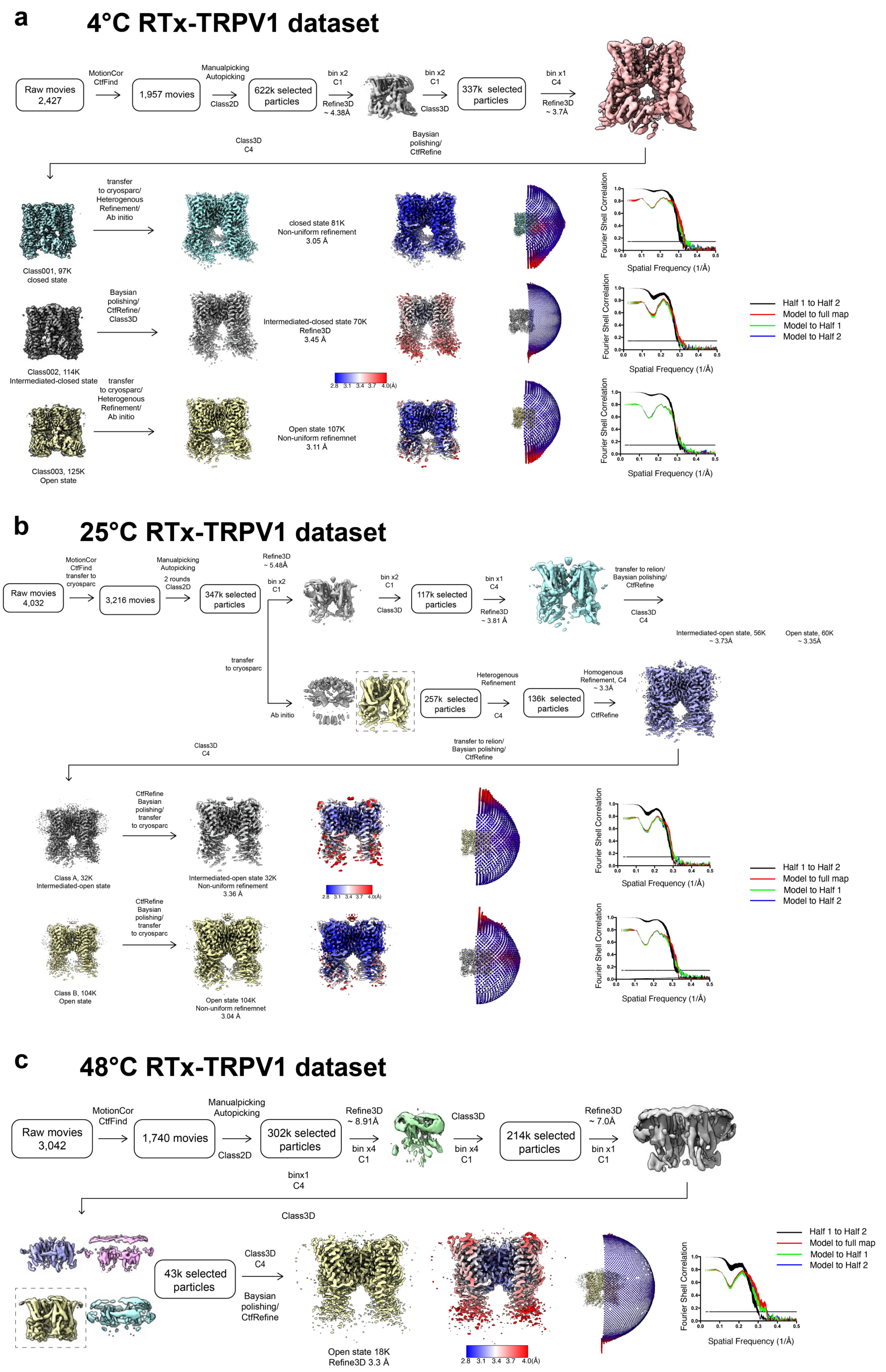

a
4°C RTx-TRPV1 dataset
Raw movies 2,427
MotionCor CtfFind
1,957 movies
Manualpicking Autopicking
Class2D
622k selected particles
bin x2 C1
Refine3D ~ 4.38Å
bin x2 C1
Class3D
337k selected particles
bin x1 C4
Refine3D ~ 3.7Å
Class3D C4
Baysian polishing/ CtfRefine
transfer to cryosparc/ Heterogenous Refinement/ Ab initio
Class001, 97K closed state
closed state 81K Non-uniform refinement 3.05 Å
Baysian polishing/ CtfRefine/ Class3D
Class002, 114K Intermediated-closed state
Intermediated-closed state 70K Refine3D 3.45 Å
transfer to cryosparc/ Heterogenous Refinement/ Ab initio
Class003, 125K Open state
Open state 107K Non-uniform refinemnet 3.11 Å
2.8 3.1 3.4 3.7 4.0(Å)
Fourier Shell Correlation
Spatial Frequency (1/Å)
Half 1 to Half 2
Model to full map
Model to Half 1
Model to Half 2
b
25°C RTx-TRPV1 dataset
Raw movies 4,032
MotionCor CtfFind transfer to cryosparc
3,216 movies
Manualpicking Autopicking 2 rounds Class2D
347k selected particles
Refine3D ~ 5.48Å
bin x2 C1
bin x2 C1
Class3D
117k selected particles
bin x1 C4
Refine3D ~ 3.81 Å
transfer to relion/ Baysian polishing/ CtfRefine
Class3D C4
Intermediated-open state, 56K ~ 3.73Å
Open state, 60K ~ 3.35Å
transfer to cryosparc
Ab initio
257k selected particles
Heterogenous Refinement
C4
136k selected particles
Homogenous Refinement, C4 ~ 3.3Å
CtfRefine
transfer to relion/ Baysian polishing/ CtfRefine
Class3D C4
CtfRefine Baysian polishing/ transfer to cryosparc
Class A, 32K Intermediated-open state
Intermediated-open state 32K Non-uniform refinement 3.36 Å
2.8 3.1 3.4 3.7 4.0(Å)
CtfRefine Baysian polishing/ transfer to cryosparc
Class B, 104K Open state
Open state 104K Non-uniform refinemnet 3.04 Å
Fourier Shell Correlation
Spatial Frequency (1/Å)
Half 1 to Half 2
Model to full map
Model to Half 1
Model to Half 2
c
48°C RTx-TRPV1 dataset
Raw movies 3,042
MotionCor CtfFind
1,740 movies
Manualpicking Autopicking
Class2D
302k selected particles
Refine3D ~ 8.91Å
bin x4 C1
Class3D
bin x4 C1
214k selected particles
Refine3D ~ 7.0Å
bin x1 C1
binx1 C4
Class3D
43k selected particles
Class3D C4
Baysian polishing/ CtfRefine
Open state 18K Refine3D 3.3 Å
2.8 3.1 3.4 3.7 4.0(Å)
Fourier Shell Correlation
Spatial Frequency (1/Å)
Half 1 to Half 2
Model to full map
Model to Half 1
Model to Half 2

Supplementary Figure 1. TRPV1 data collection and processing.

a-c Cryo-EM data processing procedures for 4°C RTx-TRPV1, 25°C RTx-TRPV1, and 48°C RTx-TRPV1 datasets, respectively. 3D reconstructions, local resolution estimation, the Euler distribution plot, and FSC curves are shown for TRPV1^{C,RTx}, TRPV1^{IC,RTx}, TRPV1$^{O,RTx(4°C)}$, TRPV1^{IO,RTx}, TRPV1$^{O,RTx(25°C)}$ and TRPV1$^{O,RTx(48°C)}$, respectively.

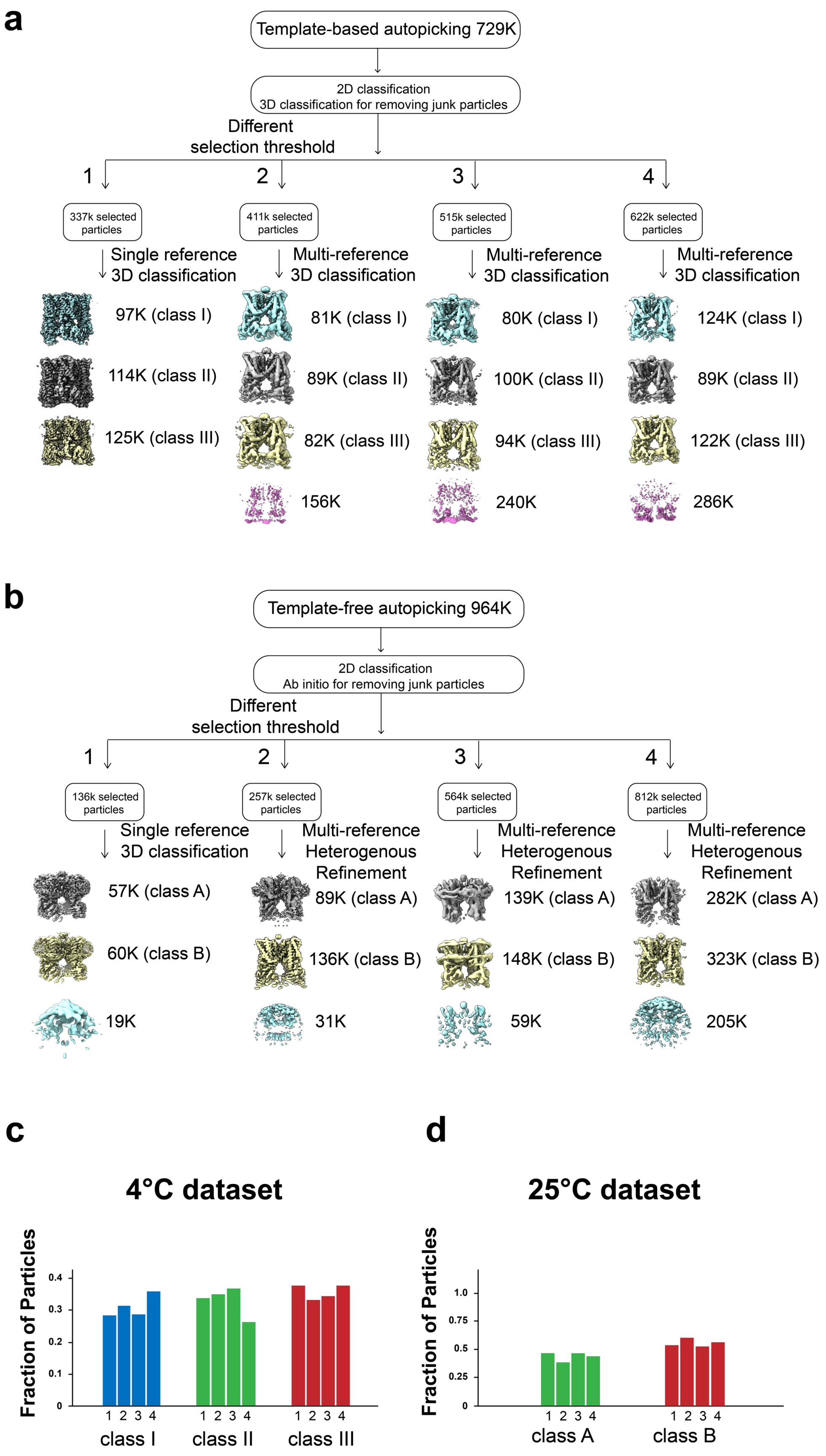
a
Template-based autopicking 729K
2D classification
3D classification for removing junk particles
Different
selection threshold
1
2
3
4
337k selected particles
411k selected particles
515k selected particles
622k selected particles
Single reference
3D classification
Multi-reference
3D classification
Multi-reference
3D classification
Multi-reference
3D classification
97K (class I)
114K (class II)
125K (class III)
81K (class I)
89K (class II)
82K (class III)
156K
80K (class I)
100K (class II)
94K (class III)
240K
124K (class I)
89K (class II)
122K (class III)
286K
b
Template-free autopicking 964K
2D classification
Ab initio for removing junk particles
Different
selection threshold
1
2
3
4
136k selected particles
257k selected particles
564k selected particles
812k selected particles
Single reference
3D classification
Multi-reference
Heterogenous
Refinement
Multi-reference
Heterogenous
Refinement
Multi-reference
Heterogenous
Refinement
57K (class A)
60K (class B)
19K
89K (class A)
136K (class B)
31K
139K (class A)
148K (class B)
59K
282K (class A)
323K (class B)
205K
c
4°C dataset
Fraction of Particles
0
0.1
0.2
0.3
0.4
1 2 3 4
class I
1 2 3 4
class II
1 2 3 4
class III
d
25°C dataset
Fraction of Particles
0
0.25
0.5
0.75
1.0
1 2 3 4
class A
1 2 3 4
class B

41

Supplementary Figure 2. Image processing workflow for the particle distribution analysis

a Data processing workflow of 4°C RTx dataset for the purpose of particle distribution analysis. **b** Data processing workflow of 25°C RTx dataset for the purpose of particle distribution analysis. **c** Class distributions of particles for the 4°C RTx dataset among independent classification procedures. **d** Class distributions of particles for the 25°C RTx dataset among independent classification procedures

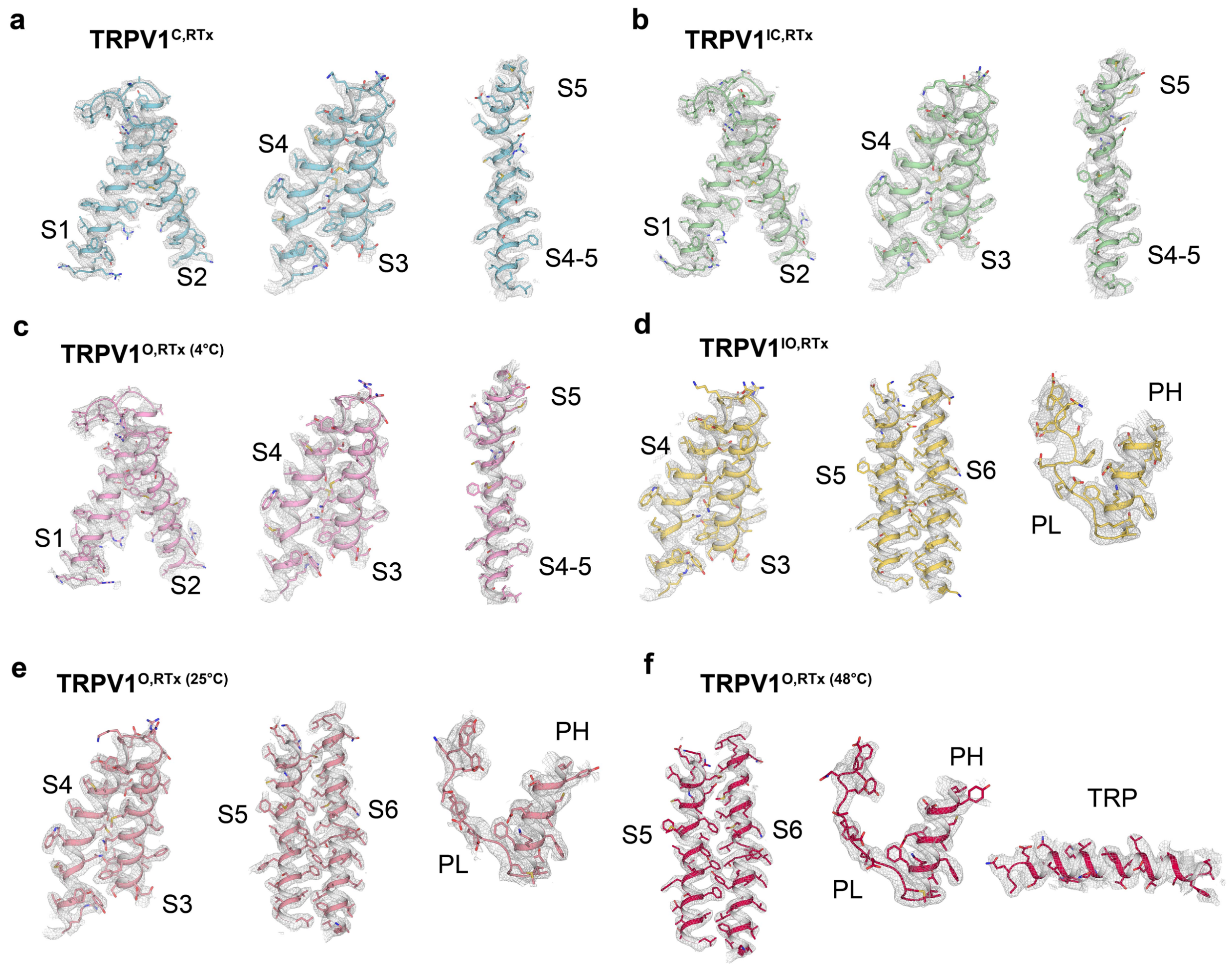

Supplementary Figure 3. Representative cryo-EM densities and models of TRPV1 from this study.

a-f Cryo-EM map for subdomains in TRPV1^{C,RTx} (**a**, thresholding 0.12), TRPV1^{IC,RTx} (**b**, thresholding 0.03), TRPV1^{O,RTx} at 4°C (**c**, thresholding 0.1), TRPV1^{IO,RTx} (**d**, thresholding 0.11), TRPV1^{O,RTx} at 25°C (**e**, thresholding 0.09), and TRPV1^{O,RTx} at 48°C (**f**, thresholding 0.033). Structural elements are shown as sticks and ribbons and EM density as gray mesh.

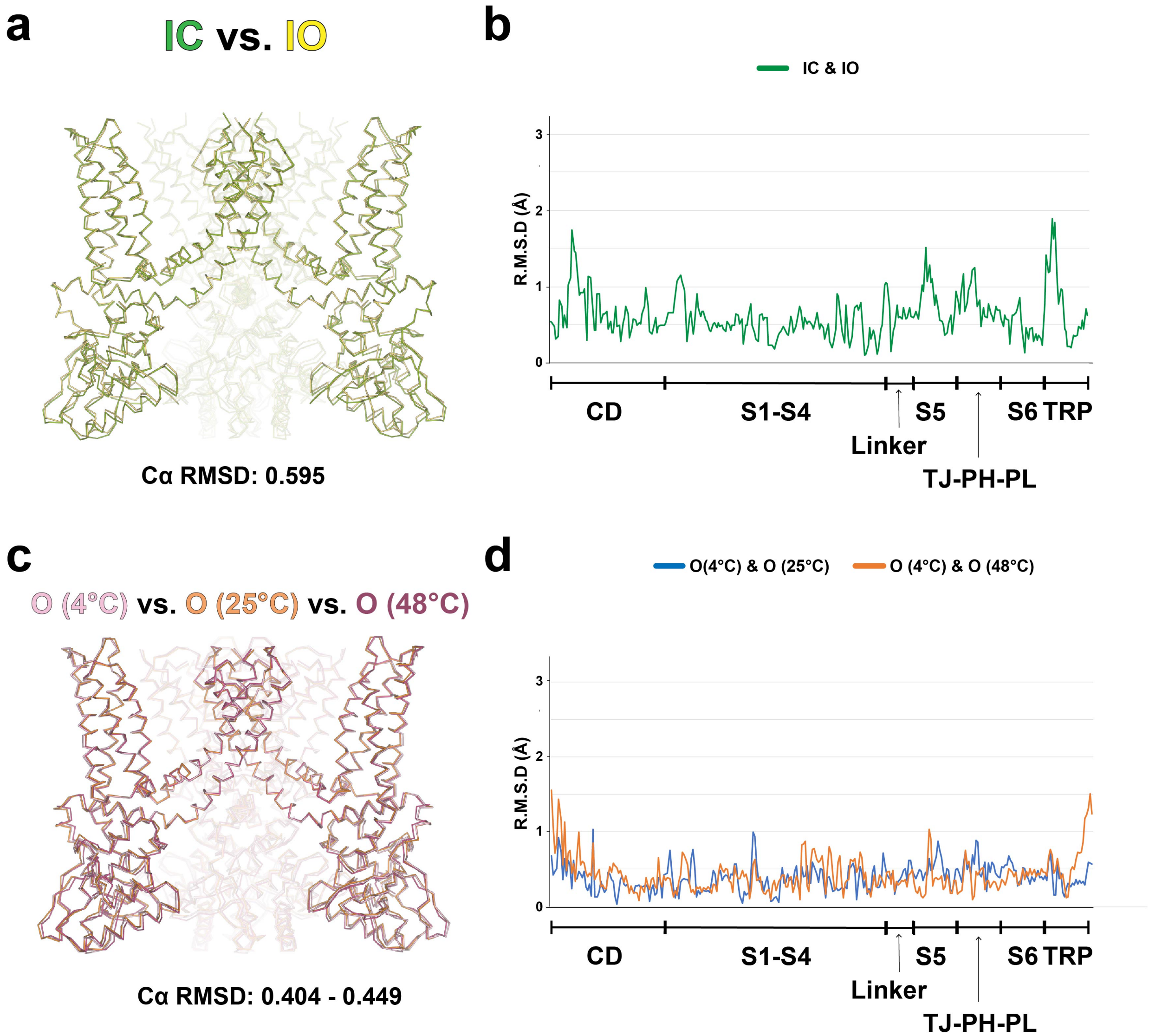

e

	C	IC	IO	O (4°C)	O (25°C)	O (48°C)
C	-	0.705	0.947	1.520	1.540	1.623
IC	0.705	-	0.595	1.132	1.140	1.217
IO	0.947	0.595	-	1.127	1.065	1.173
O (4°C)	1.520	1.132	1.127	-	0.404	0.449
O (25°C)	1.540	1.140	1.065	0.404	-	0.427
O (48°C)	1.623	1.217	1.173	0.449	0.427	-

Supplementary Figure 4. Structural similarity of TRPV1$^{\mathrm{IC,RTx}}$, TRPV1$^{\mathrm{IO,RTx}}$, TRPV1$^{\mathrm{O,RTx\,(4°C)}}$, TRPV1$^{\mathrm{O,RTx(25°C)}}$ and TRPV1$^{\mathrm{O,RTx\,(48°C)}}$

a Comparison of TRPV1$^{\mathrm{IC,RTx}}$ (green) and TRPV1$^{\mathrm{IO,RTx}}$ (gold).

b Cα R.M.S.D plot per residue between TRPV1$^{\mathrm{IC,RTx}}$ and TRPV1$^{\mathrm{IO,RTx}}$.

c Comparison of TRPV1$^{\mathrm{O,RTx(4°C)}}$ (pink), TRPV1$^{\mathrm{O,RTx(25°C)}}$ (orange) and TRPV1$^{\mathrm{O,RTx(48°C)}}$ (red).

d Cα R.M.S.D. plot per residue of TRPV1$^{\mathrm{O,RTx\,(4°C)}}$, TRPV1$^{\mathrm{O,RTx\,(25°C)}}$ and TRPV1$^{\mathrm{O,RTx\,(48°C)}}$.

e Cα R.M.S.D. values between the structures reported in this study.

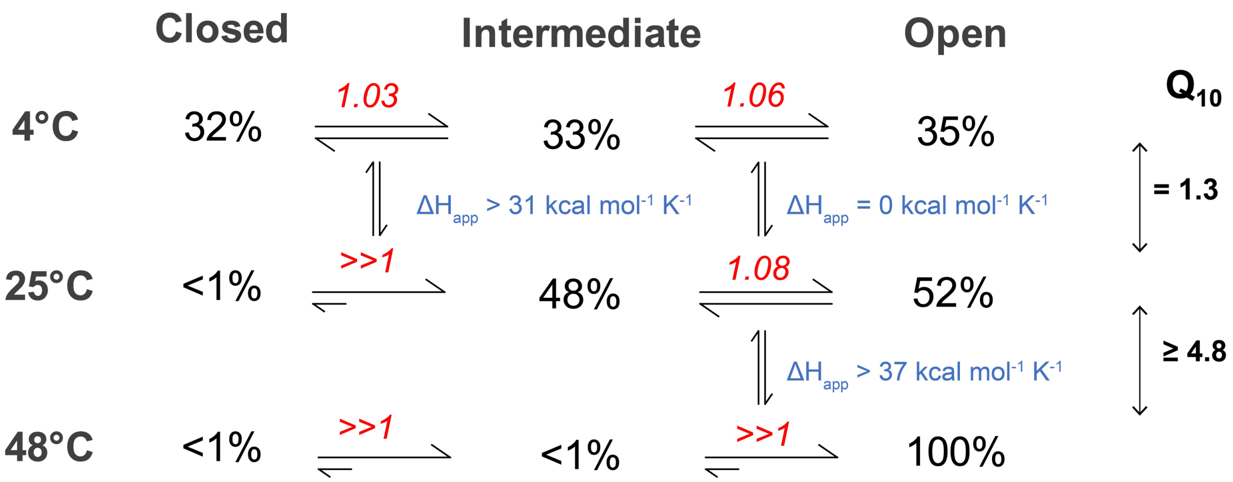

Supplementary Figure 5. Basic 3-state thermodynamic treatment of the particle distribution analysis.

Particle distribution for each state shown as percents, with calculated apparent equilibrium constants in red. Blue enthalpy values correspond to respective temperature-dependent equilibrium shifts. Apparent Q_{10} values for 4°C to 25°C and 25°C to 48°C transitions are also shown.

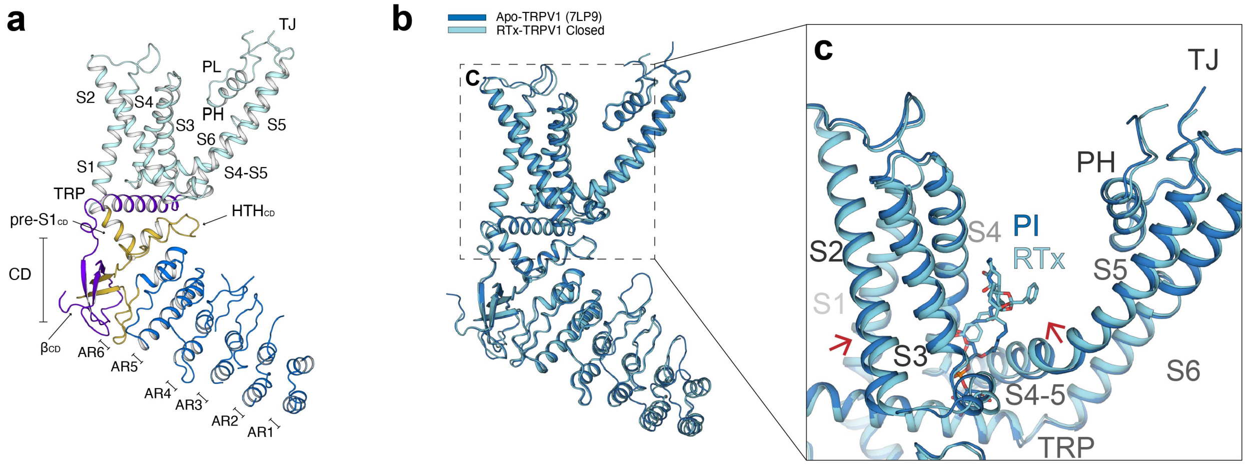

Supplementary Figure 6. Monomer architecture and structure comparison between Apo-TRPV1 and RTx-TRPV1 closed state

a Architecture of TRPV1 monomer with subdomains indicated: ankyrin-repeat domain (AR1-6), coupling domain (CD – HTHCD and βCD), transmembrane helices (S1-S6), outer-pore loop (PL), outer-pore helix (PH), turret junction (TJ), and TRP helix (TRP).

b Superposition of a single protomer from $TRPV1^{APO}$ (blue) and $TRPV1^{C,RTx}$ (skyblue).

c Close-up view of the transmembrane domain compared between $TRPV1^{APO}$ and $TRPV1^{C,RTx}$.

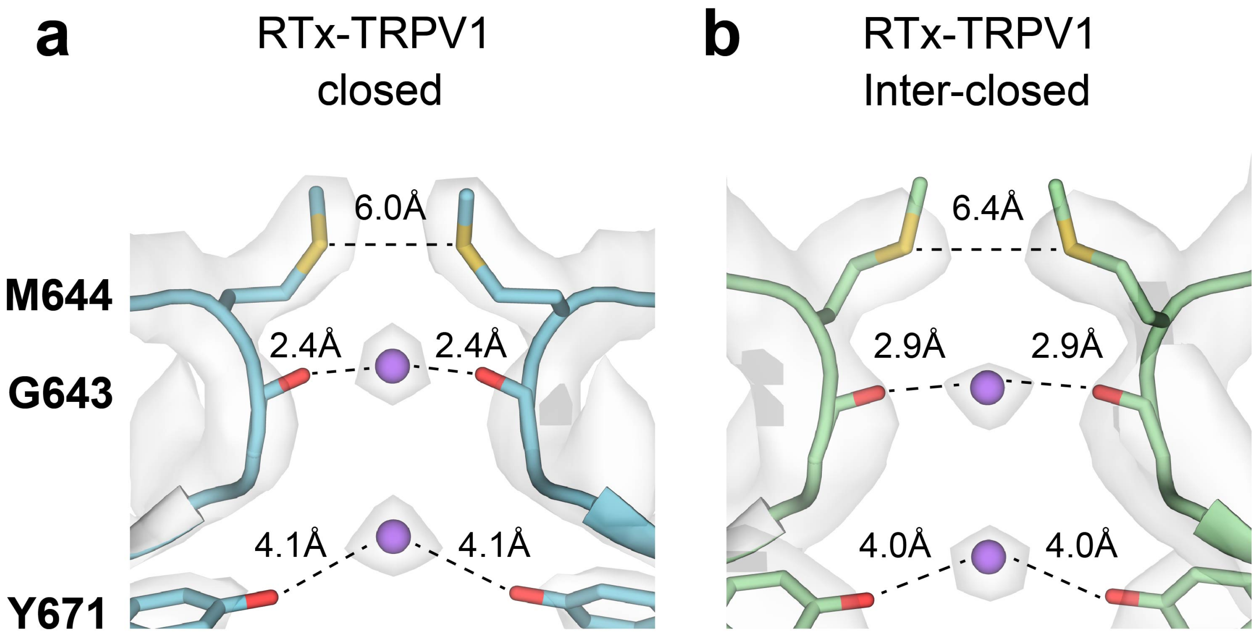

Supplementary Figure 7. Cryo-EM densities for the selectivity filter of TRPV1^{C,RTx} and TRPV1^{IC,RTx}.

a-b Cryo-EM density for the selectivity filter of TRPV1^{C,RTx} and TRPV1^{IC,RTx} corresponding to the models and putative sodium ions at 0.15 and 0.025 thresholding, respectively.

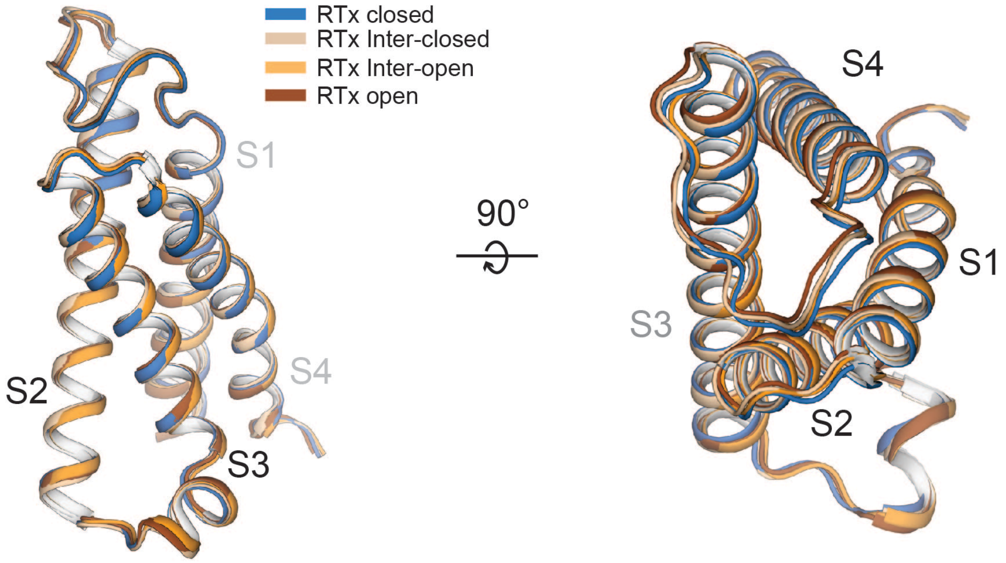

Supplementary Figure 8. The S1-S4 domain comparison of TRPV1^{C,RTx}, TRPV1^{IC,RTx}, TRPV1^{IO,RTx}, and TRPV1^{O,RTx}

Superposition of S1-S4 domain from TRPV1^{C,RTx} (blue), TRPV1^{IC,RTx} (wheat), TRPV1^{IO,RTx} (orange), and TRPV1^{O,RTx} (red)

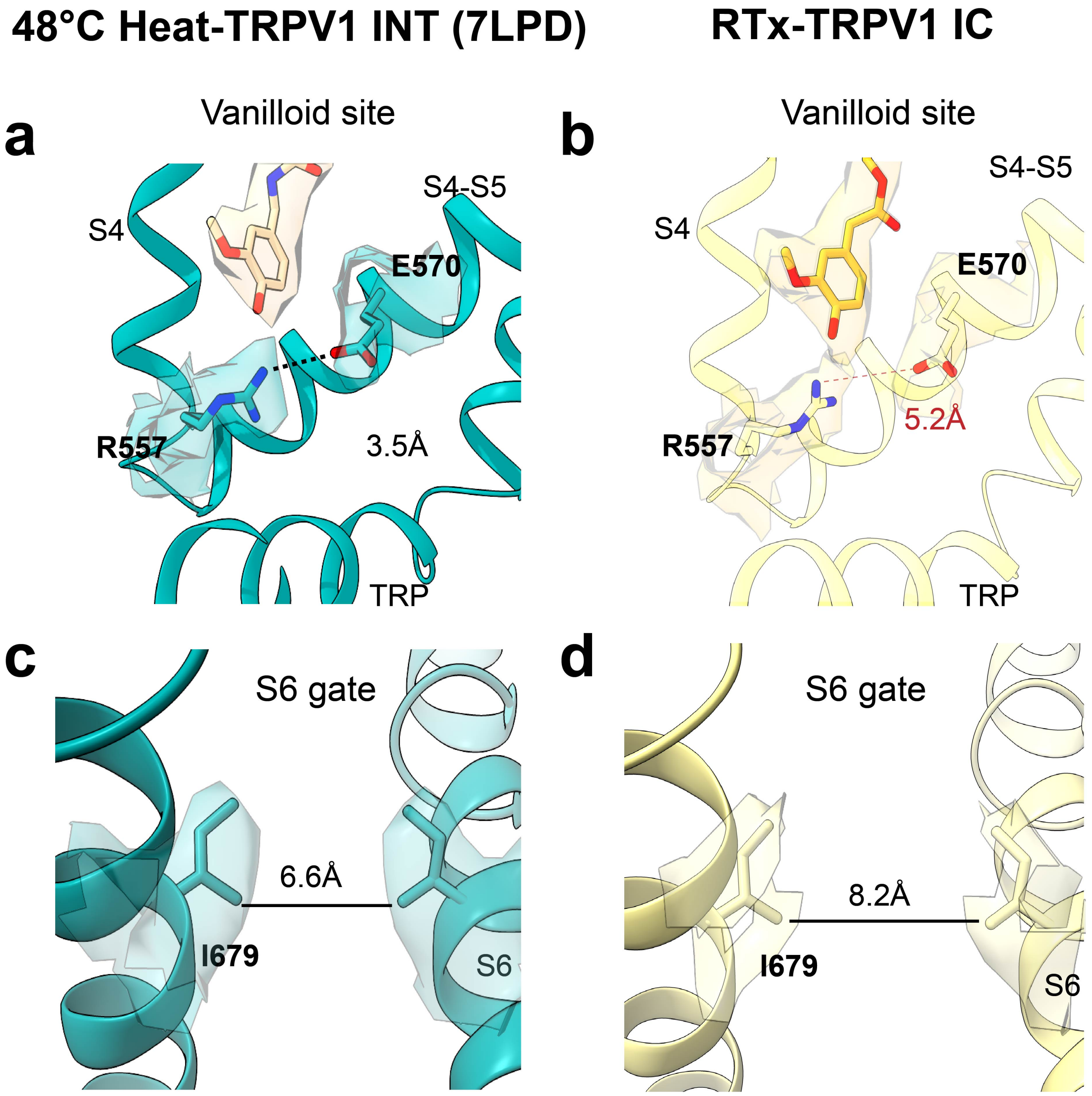

Supplementary Figure 9. Structural comparison between RTx-TRPV1 IC and Heat-TRPV1 INT.

a The cryo-EM densities and the models of the vanilloid site of TRPV1^{Heat,INT} exhibiting the salt bridge between R557 and E570, thresholding at 0.022. **b** The cryo-EM densities and the models of the vanilloid site of TRPV1^{IC,RTx}, thresholding at 0.038. Note that the salt bridge is not formed in this intermediate state. **c** The cryo-EM densities and the models of the S6 gate of of

$TRPV1^{Heat,INT}$, thresholding 0.03. **d** The cryo-EM densities and the models of the S6 gate of $TRPV1^{IC,RTx}$, thresholding 0.04.

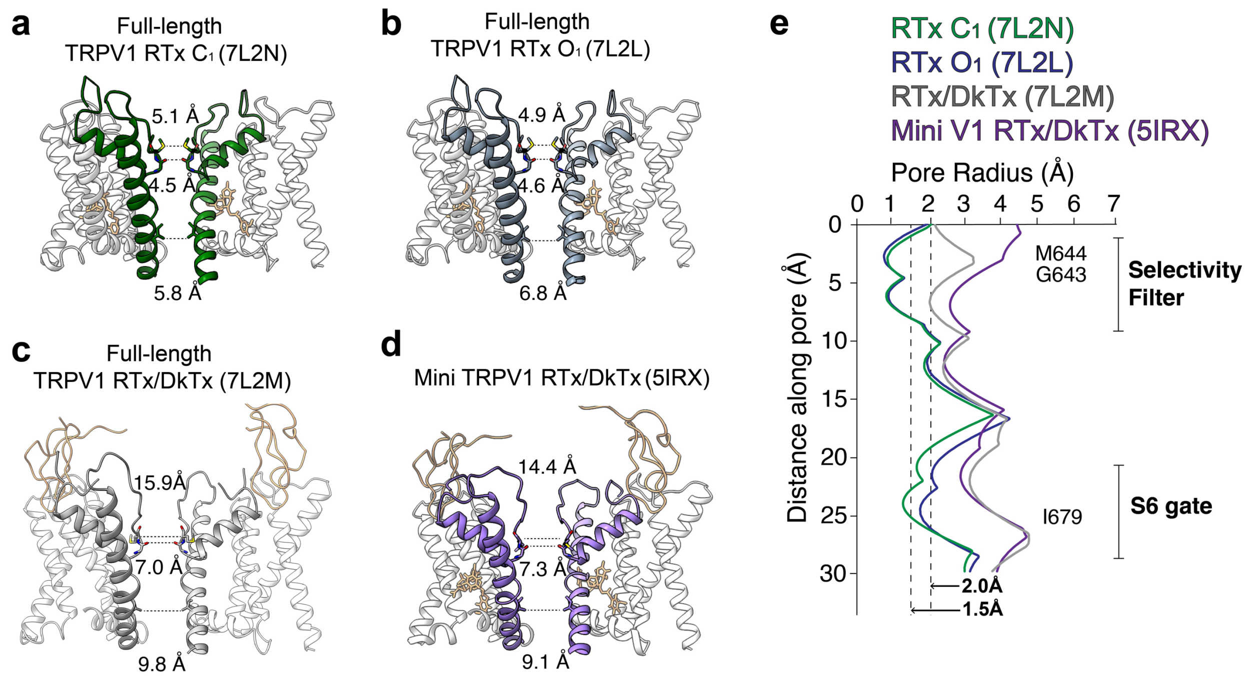

Supplementary Figure 10. Permeation pathway analysis of the pore domains of TRPV1^{RTx,C1}, TRPV1^{RTx,O1},TRPV1$^{RTx/DkTx,\ full\text{-}length}$, and TRPV1$^{RTx/DkTx,\ mini}$.

a-d Only two subunits are shown for clarity, with S6 helix (S6), pore loop (PL) and pore helix (PH) as indicated. Diagonal distances at the two narrowest restriction points are shown. Respective structure PDB codes in parentheses.

e Pore radii calculated using the HOLE program for the TRPV1 structures as color-coded

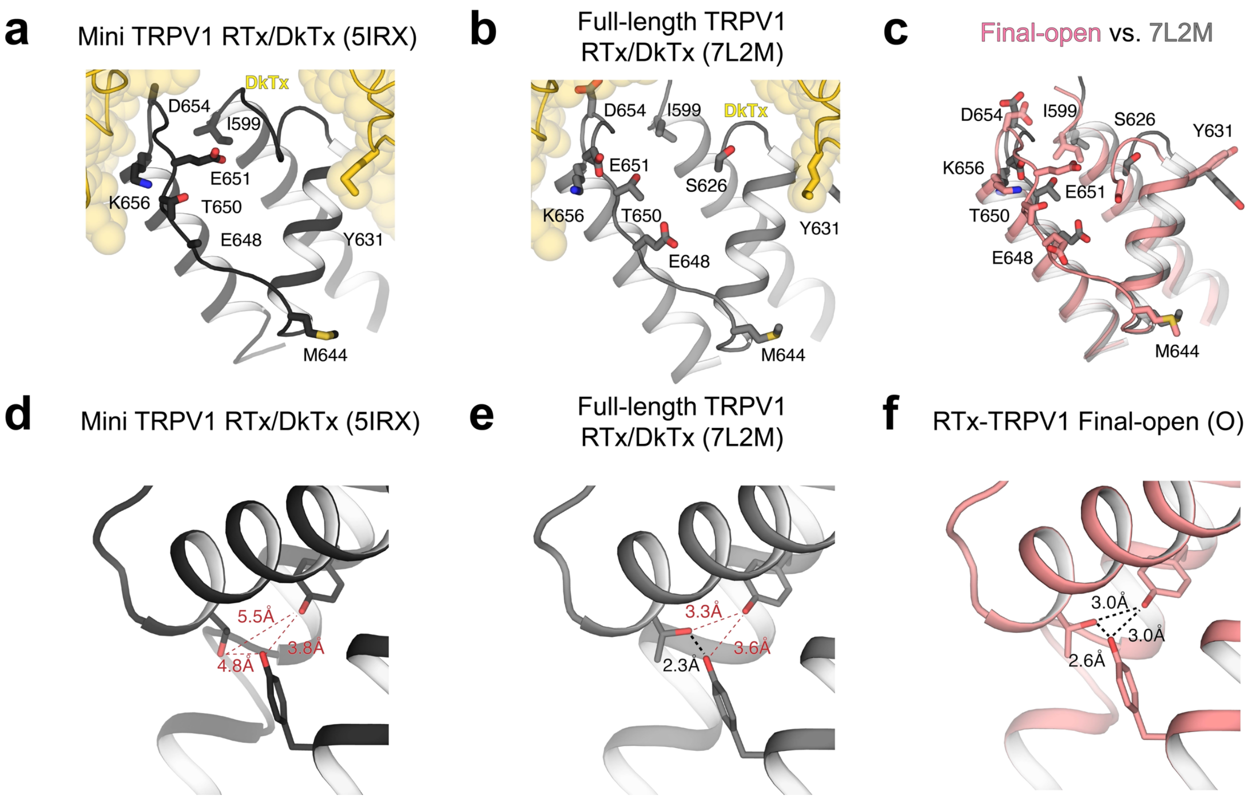

Supplementary Figure 11. The pore-loop conformation and PH-S5-S6 triad of the published full-length RTx O_1, DkTx/RTx full-length TRPV1, and DkTx/RTx mini TRPV1.

a,b Conformational comparison of $TRPV1^{DkTx/RTx,\ mini}$ and $TRPV1^{DkTx/RTx,\ full\text{-}length}$ outer pore region. Indicated residues are shown as sticks. DkTx is shown as gold sticks and spheres.

c Superposition of the outer pore region from $TRPV1^{DkTx/RTx,\ full\text{-}length}$ and $TRPV1^{O,RTx}$.

d,e,f Hydrogen bond triad networks of PH-S5-S6 of $TRPV1^{DkTx/RTx,\ mini}$, $TRPV1^{DkTx/RTx,\ full\text{-}length}$, and $TRPV1^{O,RTx}$. Black dotted-lines indicate hydrogen bonds and red lines denote distances for broken hydrogen bonds.

Supplementary Table 1. Cryo-EM data collection, refinement and validation statistics

	4 °C RTx closed (EMD- 24636, PDB 7RQU)	4 °C RTx Inter-closed (EMD-24637, PDB 7RQV)	4 °C RTx Open (EMD-24638, PDB 7RQW)	25 °C RTx Inter-open (EMD-24639, PDB 7RQX)	25 °C RTx Open (EMD-24640, PDB 7RQY)	48 °C RTx Open (EMD-24641, PDB 7RQZ)
**Data collection and processing**						
Magnification	81,000	81,000	81,000	81,000	81,000	81,000
Voltage (kV)	300	300	300	300	300	300
Electron exposure ($e^{-}/Å^{2}$)	60	60	60	44	44	50
Defocus range (μm)	-2.0 to -0.9	-2.0 to -0.9	-2.0 to -0.9	-1.9 to -0.8	-1.9 to -0.8	-1.9 to -0.8
Pixel size (Å)	1.08	1.08	1.08	1.029	1.029	1.079
Symmetry imposed	C4	C4	C4	C4	C4	C4
Initial particle images (no.)	729,895	729,895	729,895	2,679,242	2,679,242	995,671
Final particle images (no.)	81,347	70,786	107,183	32,121	104,123	18,341
Map resolution (Å)	3.05	3.45	3.11	3.36	3.04	3.32
FSC threshold	0.143	0.143	0.143	0.143	0.143	0.143
**Refinement**						
Initial model used (PDB code)	7LP9	7LP9	7LP9	7LP9	7LP9	7LP9
Model resolution (Å)	3.05	3.45	3.11	3.36	3.04	3.32
FSC threshold	0.143	0.143	0.143	0.143	0.143	0.143
Model resolution range (Å)	2.95 – 3.40	2.75 – 3.70	3.05 – 3.50	2.88 – 3.60	2.97 – 3.43	2.47 – 3.40
Map sharpening *B* factor ($Å^{2}$)	-132	-80	-140	-116	-137	-97
Model composition						
Nonhydrogen atoms	19034	18078	17176	16154	16688	17128
Protein residues	2416	2320	2180	2016	2100	2128
Ligands	38	22	20	24	16	20
B factors ($Å^{2}$)						
Protein	58.47	37.43	78.45	35.30	22.32	19.74
Ligand	29.94	15.62	28.09	18.32	7.73	9.65
R.m.s. deviations						
Bond lengths (Å)	0.005	0.005	0.006	0.003	0.005	0.004
Bond angles (°)	1.040	1.081	1.188	0.797	1.088	0.703
**Validation**						
MolProbity score	1.29	1.68	1.67	1.60	1.53	1.65
Clashscore	2.98	7.22	6.63	7.05	6.37	6.10
Poor rotamers (%)	0	0	0	0	0	0
Ramachandran plot						
Favored (%)	96.79	95.60	95.64	96.64	97.30	95.41
Allowed (%)	3.21	4.40	4.36	3.36	2.70	4.59
Disallowed (%)	0	0	0	0	0	0
